# Editorial: Methods in nanobiotechnology

**DOI:** 10.3389/fbioe.2022.1067952

**Published:** 2022-11-02

**Authors:** Piergiorgio Gentile, Chiara Tonda-Turo

**Affiliations:** ^1^ School of Engineering, Newcastle University, Newcastle Upon Tyne, United Kingdom; ^2^ Department of Mechanical and Aerospace Engineering, POLITOBioMedLAb, Politecnico di Torino, Torino, Italy

**Keywords:** nanocarriers, microrheology, computational modeling, Layer-by-layer assembly, Cryopreservation

This Research Topic collects different contributions on up-to-date methods and technologies to investigate fundamental research questions on materials at the nanoscale level and their applications in the biomedical field. Nanobiotechnology has been involved in several medical-related challenges such as smart drug delivery and gene therapy tools, nanodiagnostics, nanobiosensors, nanofluidic devices, and nanodevices. In addition, different chemical, physical and biological methods have been successfully applied in the formulation of nanomaterials, nanoparticles, and nanocolloids.

The first article of this Research Topic (Lin et al.) addresses the importance of nanotechnology-based innovations in fostering the development of therapeutic treatments for diseases that are still uncurable. This article reviewed the recent technologies proposed in the field of inner ear disorders. Indeed, nanotechnology offers several advantages as the main challenge affecting the success of inner ear treatment is the presence of physiological barriers, mainly the blood–labyrinth barrier (BLB), which limit the accessibility of the inner ear and hinder the efficacy of drugs. Smart drug delivery systems, based on nanomaterials, represent a promising strategy for drugs to reach the inner ear as nanomaterials can transport therapeutics and their surfaces can be functionalized to endow them with specific molecules able to maximize the active targeting towards auditory hair cells. Drug delivery is crucial to make a step forward in the treatment of inner ear disorders thus a more effective targeting of artificial carriers through the physiological barriers, such as the BLB, can be achieved by combining biological knowledge on barrier transport mechanisms with nanotechnologies.

The ability of nanocarriers to localize drugs into a specific tissue, reducing the risk of inactivation while circulating, can be exploited for a wide variety of medical needs. Although several methods are used for nanocarriers fabrication, the nanoscale-based technique of layer-by-layer (LbL) has demonstrated a high versatility in the development of nanocarriers for many drugs and biomolecules (Ferreira et al., 2019). Among others, LbL allows to include more therapeutical drugs and biomolecules inside on carrier in a less time-consuming manner guaranteeing a precise control of the release kinetics of each payload. In addition, both therapeutic and diagnostic cues can be included within the layers of the nanoparticles towards the design of multifunctional systems contributing to the emerging field of theranostics. Mathematical models have been proposed as powerful tools to reduce the experimental burden required to define the optimal conditions and setups to produce carriers with defined drug release profiles. Thus, Barchiesi et al. reported the development of continuum-scale mathematical model to characterize the transport and release of a drug from the shells of layer-by-layer nanofunctionalized microparticles. The developed mathematical model includes the dissolution and diffusion of the drug and accounts for a mechanism that takes into consideration the entrapment of the drug biomolecules into the polymeric shell. Authors proved the effectiveness of the computational model using the data of the release profile for an antibiotic drug, metronidazole. This article highlights the importance of a multidisciplinary approach including computational and experimental methods to evaluate the influence of changing the model conditions on the total system behaviour.

Even though nanoparticles have been applied mainly as carriers for drugs and molecules inside living organisms, the use of particles at the nanoscale level can be exploited to solve several medicine-related challenges. For instance, cryopreservation of biological samples (i.e., cells and tissues) is a delicate process as potential ice formation leads to cell damaging affecting samples preservation, therefore cryoprotective agents (CPAs) are applied to mitigate this risk. However, the CPAs are useless during the rewarming process which requires warming rates orders of magnitude above the cooling rate, without temperature gradients, to guarantee high survival of biological samples. Photothermal heating of titanium nitride nanomaterials for fast and uniform laser warming of cryopreserved biomaterials was proposed by Alvarez et al. Plasmonic nanomaterials based on titanium nitride nanomaterials successfully rewarmed cryopreserved biological systems significantly improving uniformity in heating thus becoming promising rewarming systems for next-generation cryopreservation process.

The design of novel materials and technologies requires the development of methodologies to characterize them alone and within the biological environments. As discussed by Mao et al., microrheology holds significant advantages over conventional bulk rheology, such as eliminating the need for large sample sizes, the ability to probe fragile materials non-destructively and a wider probing frequency range. The minimally invasive feature of this technology has been recently applied to investigate biomedical systems both *in vitro* and *in vivo*. Interestingly, microrheological approaches were applied to evaluate mechanical properties of cells, tissues, and biofluids exploring the heterogeneity of complex biological systems as well as monitoring the physical changes which occurred during pathologies development and progression.

The articles published in this Research Topic give an overview of potential application of nanotechnology in the biomedical sector. Fostering the knowledge at the nanoscale level is pivotal both to fabricate smart multifunctional platforms for therapies and diagnosis and to design novel computational and experimental methods to characterize complex systems.

We hope that the reader will find in this Research Topic a useful reference for the state of the art in the emerging and multidisciplinary field of methods in nanotechnology to address unsolved issues in medicine by enhancing knowledge of the biological processes at nanoscale level.
